# Rethinking health priorities for displaced populations through the integration of epigenetics and iterative reasoning

**DOI:** 10.3389/phrs.2026.1608853

**Published:** 2026-05-01

**Authors:** Alessandra Milani, Luisa Saiani, Ketti Mazzocco

**Affiliations:** 1 Department of Biomedicine and Prevention, University of Rome Tor Vergata, Rome, Italy; 2 European Institute of Oncology, IRCCS, Milan, Italy; 3 Universita Degli Studi di Verona, Verona, Italy; 4 Department of Oncology and Haemato-Oncology, University of Milan, Milan, Italy

**Keywords:** complex systems, displaced people, epigenetics, priority, trauma science

## Abstract

**Background:**

Over 120 million people are forcibly displaced worldwide, nearly half of them children. Current health responses remain largely emergency-driven, addressing immediate survival needs but overlooking the longer-term biological and psychosocial consequences of chronic trauma.

**Analysis:**

Emerging evidence suggests that severe adversities, for example those happening during pregnancy and early childhood, can become biologically embedded through changes in gene regulation. While data on humans are largely observational, and inherited transgenerational effects remain under investigation, the initial evidence reinforces the importance of prioritizing continuity of care, trauma-informed services, and early-life interventions alongside social determinants.

**Policy Options:**

We propose three complementary actions: (1) scale community-based, culturally responsive, trauma-informed mental health services integrated with primary care; (2) incorporate trauma science, epigenetics, and complex systems-related thinking into medical and social curricula; and (3) adopt iterative, adaptive policy cycles that revisit priorities through shared indicators and stakeholder feedbacks.

**Conclusion:**

Shifting from one-off crisis management to iterative, evidence-informed planning enables health systems to address both immediate needs and longer-term, potentially intergenerational risks, making responses more anticipatory, accountable, and sustainable.

## Background

The global escalation of armed conflicts and humanitarian crises has led to unprecedented levels of forced displacement. As of 2024, over 120 million individuals have been uprooted due to war, persecution, or systemic instability, representing approximately 1.5% of the global population [1]. If considered a single nation, this population would rank as the 13th largest in the world. Children constitute nearly half of all forcibly displaced, making them particularly vulnerable to the long-term consequences of displacement [2].

In this brief policy paper, the term “forcibly displaced people” refers to refugees, asylum seekers, and internally displaced persons (IDPs). Where evidence is specific to one subgroup, this is stated explicitly.

Despite the magnitude of this crisis, health responses remain predominantly reactive and short-term, focusing on emergency needs such as food, water, and shelter. These interventions, though essential, often neglect the more subtle but enduring health effects generated by chronic exposure to violence, trauma, and disrupted environments. Rodolfo Saracci [3] has coined the term “exodus hazards” to describe this layered crisis: a combination of physical, psychological, and structural stressors experienced before, during, and after forced migration [3]. These hazards stem from direct violence, prolonged uncertainty, the breakdown of familial and community ties, food insecurity, and limited access to healthcare services [4].

The cumulative nature of these hazards creates a syndemic burden that is not confined only to the displaced people. Host communities and national health systems are also deeply affected. In many receiving countries, healthcare systems are not equipped to handle the scale and intricacy of displaced populations’ needs, especially when migration is sudden or lacks sufficient international coordination, often resulting in additional burdens such as compassion fatigue or secondary trauma among host-country healthcare professionals [5]. Structural inequalities such as language barriers, cultural misalignment, and lack of legal recognition further hinder access to care [6].

Children born during displacement are especially at risk. In Ukraine, more than 1.7 million children have been displaced, many of them separated from their families and deprived of stable education or medical continuity. In Gaza, over 20,000 children have been born since the most recent conflict began, often in severely compromised perinatal conditions [7]. These children are shown to face higher risks of adverse neonatal outcomes and long-term developmental vulnerability [4, 8].

Moreover, the effects of displacement are not limited to psychosocial domains. Recent evidence from epigenetic research suggests that trauma related to forced migration can alter gene expression in ways that increase long-term susceptibility to chronic illness and mental disorders [6, 8]. While these findings support biological plausibility, much of the evidence on humans remains observational, and epigenetic markers should be interpreted as probabilistic signals rather than deterministic predictors. However, this biological embedding of trauma reinforces the need for anticipatory approaches that go beyond immediate survival and address the lasting, often invisible consequences of displacement.

In sum, current public health frameworks do not adequately capture the complexity or duration of harm associated with forced migration. A paradigm shift is needed—from short-term crisis management to long-term, integrated health planning that recognizes both the biological and systemic dimensions of displacement-related adversity.

## Analysis

The health impacts of forced displacement are wide-ranging and multi-layered, yet they remain structurally under-addressed in global and national health strategies. Existing policy frameworks typically adopt an emergency-response model that privileges acute, visible needs such as infectious disease control, sanitation, and temporary shelter. While these efforts are vital, they often fail to consider the cumulative and long-term health effects of exposure to chronic adversity, especially among children and pregnant women [4, 9, 10].

Epidemiological data show that displaced populations experience higher rates of psychological distress, including anxiety, depression, and post-traumatic stress disorder. These outcomes are strongly linked to repeated exposure to violence, family separation, prolonged uncertainty, and social exclusion [6, 11]. Structural barriers in host countries, such as limited legal status, linguistic obstacles, and lack of culturally competent care, further undermine access to essential services [5, 9, 12]. At the same time mental health disorders are associated with non-communicable diseases [6, 13].

In recent years, epigenetic research has provided converging evidence that severe or chronic trauma, especially during sensitive developmental windows such as gestation and early childhood, can become biologically embedded through changes in gene regulation (including DNA methylation). In studies on humans, these epigenetic markers are associated with long-term alterations in stress-related neuroendocrine and immune pathways, although causal inference is often constrained by observational designs and context-specific exposures [4, 6, 10, 14]. Rivera et al. [8] demonstrated that young Rwandan adults conceived through genocidal rape exhibited altered methylation patterns in genes regulating serotonin transport and neural plasticity, with corresponding elevations in anxiety and depression [8]. Importantly, the postnatal environment played a moderating role: stable caregiving and reduced exposure to adversity were associated with reduced biological risk. This finding underscores the intersection between social conditions and biological vulnerability. These insights converge with earlier research on Holocaust survivors and their descendants, where trauma-related methylation changes were observed in stress-regulation genes such as NR3C1 and FKBP5 [15].

Together, these studies support the biological embedding of trauma and are consistent with possible intergenerational effects. However, evidence for inherited transgenerational transmission in humans remains under active investigation and should be distinguished from intergenerational pathways mediated by shared environments, caregiving conditions, and social adversity [10, 14, 15]. Furthermore, projections indicate a significant rise in mental health outcomes and non-communicable diseases with congruent increases in healthcare costs [16]. Despite these findings, current public health systems rarely incorporate such perspectives into their response plans. Mental health services remain underfunded, and few countries have implemented trauma- or epigenetically-informed frameworks for displaced populations. Moreover, medical education often lacks training in areas such as psychoneuroendocrinoimmunology, developmental trauma, and sociogenomics, leaving healthcare professionals ill-equipped to recognize or respond to the long-term needs of displaced individuals [12, 17–19].

The complexity of these challenges calls for a shift in policy thinking. Rather than viewing displacement as a discrete crisis, it should be addressed as a prolonged condition with cascading effects on health systems, families, and future generations. Policymakers must therefore integrate insights from biology, psychology, and complex systems theory to craft responses that are both evidence-based, anticipatory and predictive.

## Policy options

To address the long-term health effects of forced displacement, policymakers must move beyond reactive, short-term interventions and adopt strategies that are systemic, anticipatory, predictive and rooted in interdisciplinary evidence [9]. Below is a proposal about three policy options grounded in current research and adapted to the realities of displaced populations and host countries.

1. Restructure Mental Health Services Through Community-Based, Culturally Sensitive Models

Displaced populations face significant psychological distress, often compounded by precarious living conditions and cultural disconnection. Yet, mental health services are frequently underfunded or inaccessible due to bureaucratic, linguistic, or social barriers [12].

A promising approach involves scaling up community-based mental health programs that prioritize psychosocial support, peer-to-peer interaction, and trauma-informed care. These programs should be culturally tailored and integrated into primary care settings, allowing for early identification and treatment of stress-related disorders.

How to implement the restructured Mental Health Services (examples of a two-level operational model). Community-based trauma-informed services can be embedded within existing primary care clinics, community centres, and reception facilities rather than created as parallel structures.

*First level:* at first contact, a trained community nurse or primary care nurse can conduct a brief psychosocial screening using validated instruments for distress and trauma-related symptoms. In parallel, a social worker can assess social vulnerability and support needs, including barriers related to legal status, housing, family reunification, or access to welfare and refugee recognition procedures.

*Second level:* Based on predefined thresholds and an allocation algorithm developed by clinical psychologists and used by the primary nurse or the social worker in the first level, individuals presenting with moderate or high levels of distress should be referred for the second-level clinical assessment conducted by psychotherapists or clinical psychologists.

This focused triage evaluation determines the appropriate therapeutic pathway. Depending on clinical needs, options may include structured group psychotherapy, trauma-focused individual or group therapy, outpatient psychiatric consultation for pharmacological management, or, in cases of severe risk such as suicidality, psychosis, or acute instability, referral for intensive or inpatient care. Individuals with mild distress can be supported at first level through psychoeducation, peer-support groups, stress-management interventions, and community-based psychosocial activities, with periodic reassessment to detect deterioration.

Mobile multidisciplinary teams can extend outreach to temporary shelters or informal settlements to ensure continuity of care. Governance responsibility may remain with municipal health authorities or ministries of health, working in coordination with NGOs, UN agencies, primary care networks, and trained cultural mediators and peer supporters drawn from displaced communities. Regular case-review meetings and structured supervision should be established to protect provider wellbeing and maintain quality of care.

Screening thresholds, referral criteria, and service capacity should be formally documented and reviewed periodically within the broader iterative planning framework described above.

Minimum monitoring indicators (examples):

- Coverage: proportion of forcibly displaced people screened for distress or trauma symptoms in primary care.
- Access: median waiting time for first psychosocial contact (or % seen within 2 weeks).
- Continuity: proportion completing a brief intervention or successfully referred to specialized care.

Potential impact:

- Reduces long-term burden on health systems
- Enhances social integration and recovery
- Reduces intergenerational risk pathways associated with trauma (e.g., through caregiver support and early intervention)
- Reduces the costs for the individual and the healthcare system

Barriers:

- Lack of trained personnel
- Political resistance to invest in services for forcibly displaced people
- Insufficient cooperation between health and social sectors

2. Revise Medical and Educational Curricula to Integrate Epigenetics, Trauma Science, and Psychosocial Health.

Current medical training often overlooks the biological effects of chronic trauma and the systemic nature of displacement-related illness. [8, 14]. As suggested by current literature, severe or chronic trauma can influence gene regulation (e.g., via DNA methylation), which is associated with long-term vulnerability, particularly when exposure occurs *in utero* or during early childhood [8, 14]. This paper proposes to incorporate epigenetics, psychoneuroendocrinoimmunology, and social determinants of health into the core curricula of medical, nursing, psychological, and social work programs. This would prepare future professionals to detect and respond to biologically embedded trauma in displaced individuals.

How to implement it (examples): Institutions responsible for health professional education, including accreditation bodies and universities, can incorporate educational modules across disciplines that promote a systemic understanding of the individual. Accreditation bodies could formally require the inclusion of standardized modules on trauma science, epigenetics, and social determinants of health as a condition for program approval or periodic reaccreditation. In this perspective, psychoneuroendocrinoimmunology and complexity science, together with training in trauma-informed care and social determinants of health, would help students and health professionals integrate biological, psychological, and social dimensions of complex vulnerability. Educational strategies should include case-based teaching drawn from displaced people reception centers and primary care settings, interprofessional simulations (medicine, nursing, psychology, social work), and faculty development initiatives with continuing education and structured support for host-country provider wellbeing.

Minimum monitoring indicators (examples):

- Program adoption: number or proportion of training programs including a standardized module on trauma science and epigenetics.
- Competency: pre/post knowledge or skills assessment in trauma-informed and culturally safe care.
- Workforce wellbeing: uptake of supervision/support resources among frontline providers.

Potential impact:

- Builds health system capacity for long-term care
- Promotes early, preventive interventions
- Supports interprofessional collaboration
- Address the wellbeing of host-country healthcare professionals

Barriers:

- Institutional inertia in education systems
- Need for faculty retraining and curriculum development
- Limited awareness of emerging biological and psychological research

3. Implement Iterative, System-Based Planning Frameworks in Health Policy.

**FIGURE 1:**
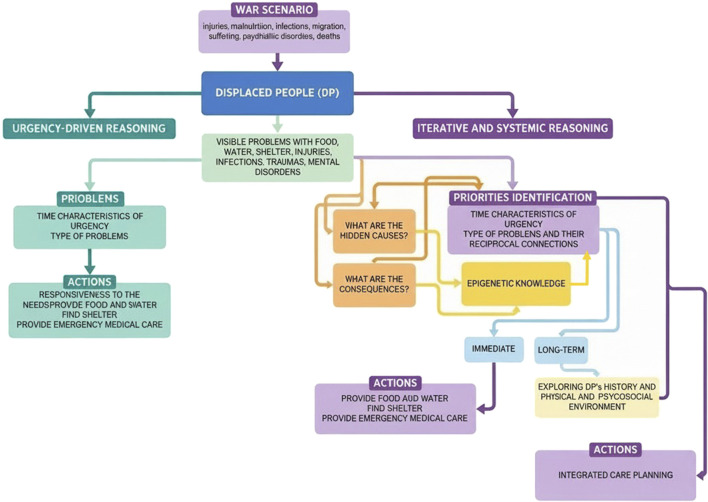
Dual priority-setting logic in war-related public health interventions (Author elaboration with AI support, 2026).

Traditional health planning often relies on linear reasoning and short-term metrics, focusing healthcare priorities on urgency-driven factors. However, the complex, interdependent nature of displacement harms calls for iterative reasoning. In psychology, iterative reasoning is defined as “a reasoning strategy wherein the initial conclusion suggested by a set of premises is integrated into that set of premises in order to yield additional conclusions”(20). Building on this conceptual foundation, we define iterative reasoning in health policy as a structured cycle of priority-setting in which institutions (I) define explicit goals and time horizons (acute, medium-term, long-term), (II) select indicators that capture both visible and latent harms (e.g., service continuity, school attendance, caregiver distress, trauma symptom screening, and early markers linked to chronic disease risk), (III) implement interventions with clear learning objectives, (IV) monitor outcomes and contextual changes, and (V) revise premises and consequent priorities and resource allocation at pre-specified intervals based on updated data and stakeholder feedback [20, 21]. In this model, urgency is treated as one interacting variable rather than the sole decision rule, reducing the risk that salient short-term crises crowd out prevention and continuity of care [18]. Evidence from clinical prioritization research shows that when decision-makers face incongruent cues, prioritization accuracy can collapse due to predictable cognitive biases, reinforcing the value of explicit update-and-review cycles rather than one-shot, salience-driven choices [22]. As illustrated in Figure 1, the urgency-driven pathway follows a linear, one-shot logic, whereas the iterative pathway incorporates predefined review cycles and feedback loops. Box 1 provides a practical case illustration of how this iterative approach can be applied to the maternal–child and adolescent health continuum within a host-city reception system.

The diagram contrasts two models of decision-making following war-related displacement. The left pathway represents an urgency-driven logic focused on immediately visible problems (e.g., food, water, shelter, acute injuries), leading to rapid, one-directional action. The right pathway illustrates a systemic, iterative reasoning approach that integrates short-, medium-, and long-term considerations. This model incorporates reflection on hidden causes, downstream consequences, and evolving evidence (psychosocial and epigenetic) insights to inform priority identification and periodic reassessment. While both pathways include immediate emergency responses, the iterative model introduces cyclical review and multi-level planning, enabling adaptive and integrated care strategies over time. Source: Author elaboration with AI support, 2026.

BOX 1 **Case Illustration - start here.**

A practical example is the maternal child and adolescent health continuum within a large host-city reception system. In the first 0–3 months, priorities may focus on safe delivery pathways, vaccination catch-up, nutrition support, acute mental health triage, and protection services for unaccompanied minors. After 6–12 months, a pre-defined dashboard (e.g., antenatal/postnatal follow-up, service uptake, repeated emergency visits, school attendance, and screening results for trauma-related symptoms) can trigger a revision that strengthens community parenting support, school-linked psychosocial services, and culturally appropriate trauma-focused interventions for adolescents, alongside continuity pathways for chronic disease prevention. Over time, medium- and long-horizon signals, including emerging evidence consistent with biological embedding of stress (with epigenetic findings interpreted as risk indicators rather than deterministic predictors, and with human evidence still largely observational and context-dependent), can support sustained protective environments and continuity of care, while the intervention package is updated as evidence and constraints evolve [18–20].

Policy tools should be developed to support adaptive planning, allowing institutions to simulate how interventions in one domain (e.g., early childhood care) affect outcomes across others (e.g., educational attainment, chronic disease rates). This would align with the Adaptive Healthcare Organization (AHO) model proposed by Carter and Burke [23], which emphasizes real-time decision-making and responsiveness to frontline data [9, 23].

How to implement (examples). Establish a joint priority-setting group at the appropriate governance level (e.g., city health authority or ministry unit) with mandated participation from primary care, maternal–child health, mental health, education/protection services, NGO/UN partners, and representatives of forcibly displaced communities. Agree on a small dashboard of indicators, run quarterly reviews early on, and link each review to explicit decisions on resources, responsibilities, and timelines. Use rapid learning pilots (e.g., 6–12 weeks) when evidence is uncertain.

Minimum monitoring indicators (examples):

- Continuity: proportion with a documented primary-care contact and follow-up plan after 6 months.
- Maternal–child: antenatal/postnatal follow-up coverage and school attendance among children/adolescents.
- System pressure: repeated emergency visits or avoidable hospitalisations in the displaced catchment area.

Potential impact:

- Enhancing policy resilience and adaptability
- Reducing unintended consequences of isolated interventions
- Encouraging ethical, long-term accountability

Barriers:

- Requiring interdepartmental coordination
- Demanding data infrastructure and skilled analysts
- Possible conflict with short-term political cycles

These three options are mutually reinforcing. Together, they support a shift from emergency response to sustainable, biologically-informed public health systems capable of addressing both immediate and generational consequences of displacement.

## Conclusion

Forced displacement is a profound public health challenge that extends far beyond the provision of food, shelter, and emergency care. As the evidence shows, its effects are interwoven into the biological, psychological, and social fabric of individuals and communities—often persisting across generations. Traditional health responses, while necessary in the immediate aftermath of displacement, are insufficient to address the deeper and more enduring forms of harm associated with what Saracci [3] calls “exodus hazards” [3].

This Policy Brief links two complementary lenses for rethinking health priorities for forcibly displaced people. First, epigenetic research and related trauma science suggest that severe or chronic adversity, especially during pregnancy and early childhood, are associated with biological embedding through changes in gene regulation, with implications for later mental and physical health. In humans, evidence is largely observational and context-dependent, and putative inherited effects remain under investigation. Second, iterative reasoning translates this long-horizon perspective into practice by treating priority setting as a structured review cycle rather than a one-off, urgency-only decision [18–20]. Together, these lenses support the three policy options outlined in this brief: trauma-informed community mental health; curriculum reform; and adaptive, indicator-driven planning.

Implementing these options requires political commitment, cross-sector governance, and investment in workforce capacity and data infrastructure. Adaptive models such as the Adaptive Healthcare Organization emphasise that resilience comes from learning systems that use frontline data to update decisions, not from rigid protocols [21].

Ultimately, responding to forced displacement means addressing not only immediate survival needs but also the slower, often less visible pathways to lifelong inequality and illness. A cautious, non-deterministic use of epigenetic insights, combined with iterative, accountable decision cycles, can help health systems protect health across the life course and reduce preventable downstream harms.
